# Sex-dependent right ventricular hypertrophic gene changes after methamphetamine treatment in mice

**DOI:** 10.1016/j.ejphar.2021.174066

**Published:** 2021-06-05

**Authors:** Hicham Labazi, Margaret Nilsen, Margaret R. MacLean

**Affiliations:** a Institute of Cardiovascular & Medical Sciences and College of Medical, Veterinary & Life Sciences, University of Glasgow, Glasgow, UK; b Strathclyde Institute of Pharmacy and Biomedical Sciences, University of Strathclyde, Glasgow, UK

**Keywords:** Methamphetamine, pulmonary arterial hypertension, Right ventricular hypertrophy, Sexual dimorphism

## Abstract

Methamphetamine (MA) abuse is associated with the development of pulmonary arterial hypertension (PAH) and subsequent right ventricular failure. A recent clinical study demonstrated that female sex is a major risk factor for MA-induced PAH. The mechanisms associated with increased prevalence and severity of MA-induced PAH in females are still unclear. We hypothesized that MA may promote changes in gene expression in the right ventricle contributing to the development and/or worsening of PAH in females. Male and female C57BL/6 mice were treated with either MA or vehicle. Right and left ventricular systolic pressures (RVSP and LVSP, respectively) were assessed and tissue samples were collected for gene expression and histology. LVSP and RVSP were not affected by MA in either males or females. Right ventricular hypertrophy was significantly increased by MA in females but it was not affected by MA in males. In the female mice, MA-induced right ventricular hypertrophy was associated with increased expression of brain natriuretic peptide gene and members of the TGF-β receptor signaling pathway such as TGF-β receptor-1, smad3 and smad7. In male mice, there were no changes in right ventricular gene expression. Our results suggest that MA caused right ventricular hypertrophy in female mice, but not in males and that this was associated with an increase in hypertrophic genes. The right ventricular hypertrophy was not dependent on increased RVSP suggesting a direct effect of MA on the right ventricle. If this translates to PAH patients, it might explain the poor outcome observed in MA-associated female PAH patients.

## 1 Introduction

Methamphetamine (MA) is a highly addictive psychostimulant drug and can be detrimental psychologically, medically and socially. Abusers of MA are more likely to develop neurological diseases such as depression, schizophrenia and psychosis (Yang et al., 2018). In addition to these neurological effects, MA use is associated with cardiovascular complications including cardiac arrhythmias, stroke, cardiomyopathy and pulmonary arterial hypertension (PAH) (Ho et al., 2009; Huang et al., 2016; Lappin et al., 2017; Wijetunga et al., 2003; Zamanian et al., 2018; Zhao et al., 2018). PAH is a life-threatening disease characterized by remodeling of small pulmonary arteries, increased pulmonary artery pressure and pulmonary vascular resistance, leading to hypertrophy and eventual fatal failure of the right ventricle (RV). Chin et *al.* reported that MA abuse significantly increases the risk of developing PAH. Recent retrospective studies and a prospective cohort studies have also reported that MA-induced PAH (MA-PAH) is severe and progressive with poor outcomes (Zamanian et al., 2018; Zhao et al., 2018). It was suggested by the WHO that MA should be upgraded from a “likely” risk factor for PAH to a “definite” risk factor (Ramirez et al., 2018; Simonneau and Humbert, 2018). Interestingly, in their retrospective analysis of MA users, Zhao et al. reported that female sex was the only risk factor associated with MA-PAH (Zhao et al., 2018). This is consistent with the prevalence of PAH being higher in females (Franco et al., 2019; Zhao et al., 2018). Experimentally, mice treated with MA exposed to hypoxia exhibit pulmonary artery remodeling associated with mitochondrial dysfunction and DNA damage (Chen et al., 2017a). Unfortunately, only male rodents have been used to study potential mechanisms that may contribute to MA-associated PAH, despite up to 4-fold more women developing PAH (Frost et al., 2011). Furthermore, Rodent studies (Milesi-Halle et al., 2007; Ohia-Nwoko et al., 2017; Schindler et al., 2002) and a recently published human study (Mayo et al., 2019) demonstrate that females are more sensitive to psychomotor-activating effects of MA than male, and that sex should be considered when assessing behavioral responses to MA. MA also exerts cardiac toxicity and dysfunction by modulating cardiomyocyte cellular signaling (i.e. increased calcium entry and apoptosis) (Chen et al., 2016; Liang et al., 2010; Sugimoto et al., 2009). Interestingly, recent studies have shown that prenatal and adult exposure to MA resulted in a larger infarct size in response to ischemia-reperfusion in female rats, with no effect on male hearts, suggesting a hypersensitivity of female heart to ischemic injury (Rorabaugh et al., 2016, 2017). In light of the evidence, our hypothesis was that MA may promote changes in gene expression in the heart and the lung contributing to the development and/or worsening of PAH in females when compared in males. In the present study, we examined expression of genes which are known to influence the pathogenesis of PAH such as genes associated with fibrosis, hypertrophy and vascular remodeling.

## 2 Materials and methods

### 2.1 Animals

All experimental procedures were carried out in accordance with the United Kingdom Animal Procedures Act (1986) and with the “Guide for the Care and Use of Laboratory Animals” published by the US National Institutes of Health (NIH publication No. 85–23, revised 1996), and ethical approval was also granted by the University of Glasgow and University of Strathclyde Ethics Committees. All procedures were performed under the UK Home Office establishment licence number X56B4FB08 awarded to The University of Strathclyde.

*Mice:* 9–10 weeks old C57BL/6 female and male mice (Envigo, UK) were treated twice a day, 5 days/week for 3 weeks with either 0.5 mg/kg Methamphetamine (Sigma-Aldrich, UK) or vehicle (0.2–0.3% methanol in PBS). Mice were weighted before injections. Mice were housed in a 12-h light dark cycle with access to food and water ad libitum.

### 2.2 In vivo assessment of pulmonary hypertension

For all in vivo procedures, mice were anesthetized with inhaled isoflurane (3% in O_2_, induction; 1–1.5% in O_2_, maintenance), the level of anesthesia was assessed by absence of pedal reflex to toe pinch. In vivo pressure–volume loop relation measurements were performed to assess hemodynamic alterations in anesthetized mice after 3 weeks of treatment with MA or vehicle. A pressure catheter (Millar Instruments, Houston, TX) was inserted into the left ventricle (LV) via the carotid artery and to the right ventricle (RV) via the right jugular vein. After stabilization, steady-state measurements were recorded. LV and RV systolic pressure (LVSP and RVSP *respectively*), heart rate (HR) and ventricular contractility were evaluated. At the end of the procedure, mice were killed by exsanguination under terminal anesthesia (5% isoflurane), and lung and RV were collected for gene expression.

### 2.3 Measurement of right ventricular hypertrophy

Right ventricular hypertrophy (RVH) or Fulton index was assessed as the weight of the RV free wall/the weight of the left ventricle with the septum (Fulton Index = RVH = RV/(LV + septum)).

### 2.4 Gene expression

Lung and RV tissue were isolated from vehicle and MA-treated male and female mice and were stored at −80 °C until RNA isolation was performed. Lung and RV tissues were lysed using a TissueLyser (Qiagen). Total RNA from lung and RV mouse tissues were extracted using the QIAGEN miRNeasy mini-kit (Qiagen, Manchester, UK) following the manufacturer's instructions. Treatment with DNAse 1 (Qiagen) eliminated genomic DNA contamination prior to quantification using a NanoDrop ND-1000 Spectrophotometer (Nano-Drop Technologies, Wilmington, DE, USA). RNA was then reverse transcribed to cDNA using the TaqMan Reverse Transcription kits (Life technologies, Paisley, UK). The mRNA expression was assessed using TaqMan Gene Expression probes (Life Technologies, Paisley, UK) by quantitative real-time polymerase chain reaction (qRT-PCR) and normalized to a housekeeper. For gene expression, β-actin and β-2-microglobulin (B2M) were used for lung and RV samples, respectively. TaqMan assay ID are presented in Table 1. In the present studies we looked at the expression of genes which change of expression contribute to the pathogenesis of PAH (such as bone morphogenetic protein receptor, type II (*BMPR2*), 5-hydroxytryptamene receptor 1B *(HTR1B) and* cytochrome P450 A1 and B1 (*CYP1B1, CYP1A1)*, and markers of fibrosis (such as collagen type I and III. (*cola1a1 and col3a1)* and fibronectin (*FN1*)) in the lung, as well as the right ventricular hypertrophy and fibrosis markers (natriuretic peptide A and B (*ANP* and *BNP) and* transforming growth factor*-β (TGF-β)* signaling pathway*)*.

**Table 1:** TaqMan® gene expression assays IDs used for the gene expression experiments.

Gene name	Assay ID
Actb (beta actin)	Mm00607939_s1
Col1a1 (collagen, type I, alpha 1)	Mm00801666_g1
Cyp1b1 (cytochrome P450, family 1, subfamily b, polypeptide 1)	Mm00487229_m1
B2m (beta-2 microglobulin)	Mm00437762_m1
NPPA (natriuretic peptide type A)	Mm01255747_g1
NPPB (natriuretic peptide type B)	Mm01255770_g1
Eng (endoglin)	Mm00468256_m1
Smurf 1 (SMAD specific E3 ubiquitin protein ligase 1)	Mm00547102_m1
Smurf 2 (SMAD specific E3 ubiquitin protein ligase 2)	Mm03024086_m1
Smad 2 (SMAD family member 2)	Mm00487530_m1
Smad 3 (SMAD family member 3)	Mm01170760_m1
Smad 7 (SMAD family member 7)	Mm00484742_m1
GPER (G protein-coupled estrogen receptor 1)	Mm02620446_s1
ESR 1 [estrogen receptor 1 (alpha)]	Mm00433149_m1
ESR 2 [estrogen receptor 1 (beta)]	Mm00599821_m1
Tgfbr1 (transforming growth factor, beta receptor I)	Mm00436964_m1
Htr1b (5-hydroxytryptamine (serotonin) receptor 1B)	Mm00439377_s1
Bmpr2 [bone morphogenetic protein receptor, type II (serine/threonine kinase)]	Mm00432134_m1
Cyp1a1 (cytochrome P450, family 1, subfamily a, polypeptide 1)	Mm00487218_m1
Fn1 (fibronectin 1)	Mm01256744_m1
Col3a1 (collagen, type III, alpha 1)	Mm01254476_m1
Tgfb1 (transforming growth factor, beta 1)	Mm01178820_m1

### 2.5 Pulmonary artery remodeling and immunohistochemistry

Remodeling: 5 μm lung sagittal sections were stained with elastin/Picro Sirius red for identification of vascular remodeling. Pulmonary arteries (<100 μm in diameter) were microscopically assessed for degree of muscularisation in a blinded fashion. Remodeled arteries were confirmed by the presence of double-elastic laminae, and percentage remodeling (percent of remodeled vessels) was defined for each animal by the number of remodeled vessels divided by the total number of vessels observed in the lung (>80 vessels). One slide per mouse was visualized and analyzed. All vessels in a visual field were counted using a 40X objective. Images were captured using a Zeiss Axio Imager M1.

Immunohistochemistry: 5 μm sections of mouse lung were dewaxed and rehydrated through an ethanol gradient before antigen retrieval in citric acid buffer. Non-specific blocking was achieved using normal horse serum (2.5%) at room temperature. Proliferating cell nuclear antigen (PCNA) is a marker of proliferation. Sections were incubated with rabbit polyclonal PCNA (Abcam 1:3000 dilution) for 2 h at room temperature or alpha smooth muscle actin (Abcam 1:500 dilution) overnight at 4 °C. Anti-rabbit alkaline phosphatase polymer conjugated secondary antibody (Vector Labs mp-5401) was used and immuno-localization was visualized with a vector red substrate kit and counterstained with haematoxylin. Immunostaining was examined in at least n = 5 animals, and for each animal lung, measurements were repeated 2–3 times in different sections. For each animal, a total of 12–18 measurements were made. PCNA analysis was carried out using Image J Fiji software (v 2.1). Smooth muscle actin analysis was carried out using Zen 2 software (v 2.5).

### 2.6 Statistical analysis

Data were analyzed using Student's *t-*test for significance to compare treatment groups to vehicle controls. Data are expressed as means ± S.E.M. (n), where ‘n’ is the number of mice. Values of P < 0.05 were considered statistically significant.

## 3 Results

### 3.1 Effect of MA treatment on physiological parameters

Compared to age- and weight-matched vehicle-treated female mice, MA-treated female mice exhibited RV hypertrophy which was evident by increased RV weight, and RV index (Fulton index). Body weight, heart weight and left ventricular weight were not significantly affected by MA treatment (Table 2). In male mice, no difference was observed in physiological parameters between the vehicle and the MA-treated mice (Table 3).

**Table 2:** Changes in physiological parameters in response to Methamphetamine (MA) treatment in female mice: MA-treated mice exhibit right ventricular (RV) hypertrophy compared to vehicle-treated female mice. RV (right ventricle), LV + S (left ventricle + septum). Results are expressed as mean ± standard error of the mean (S.E.M).^a^ P < 0.05 *vs.* vehicle group.

	Vehicle (n = 6)	MA (n = 6)
Body weight, g	20.52 ± 0.49	20.77 ± 0.68
Heart weight, mg	97.70 ± 2.91	102 ± 2.31
Tibia length, mm	19 ± 0.34	18.08 ± 0.58
RV, mg	16.48 ± 0.68	19.90 ± 1.16^a^
LV + S, mg	81.22 ± 2.41	82.08 ± 2.11
RV/LV + S	0.20 ± 0.01	0.24 ± 0.02^a^
RV/tibia length, mg.mm^−1^	0.87 ± 0.03	1.10 ± 0.07^a^
LV + S/tibia length, mg.mm^−1^	4.28 ± 0.14	4.56 ± 0.20

**Table 3:** Changes in physiological parameters in response to Methamphetamine (MA) treatment in male mice: no difference was observed between the vehicle and the MA-treated male mice. RV (right ventricle), LV + S (left ventricle + septum). Results are expressed as mean ± standard error of the mean (S.E.M).

	Vehicle (n = 8)	MA (n = 8)
Body weight, g	28.04 ± 0.63	26.49 ± 0.98
Heart weight, mg	136.2 ± 9.87	121.6 ± 5.78
Tibia length, mm	17.88 ± 0.23	17.69 ± 0.19
RV, mg	25.85 ± 1.27	23.84 ± 1.25
LV + S, mg	110.3 ± 8.66	97.74 ± 4.65
RV/LV + S	0.24 ± 0.01	0.24 ± 0.01
RV/tibia length, mg.mm^−1^	1.45 ± 0.06	1.35 ± 0.06
LV + S/tibia length, mg.mm^−1^	6.15 ± 0.44	5.52 ± 0.24

### 3.2 Effect of MA treatment on RVSP

**Fig. 1:**
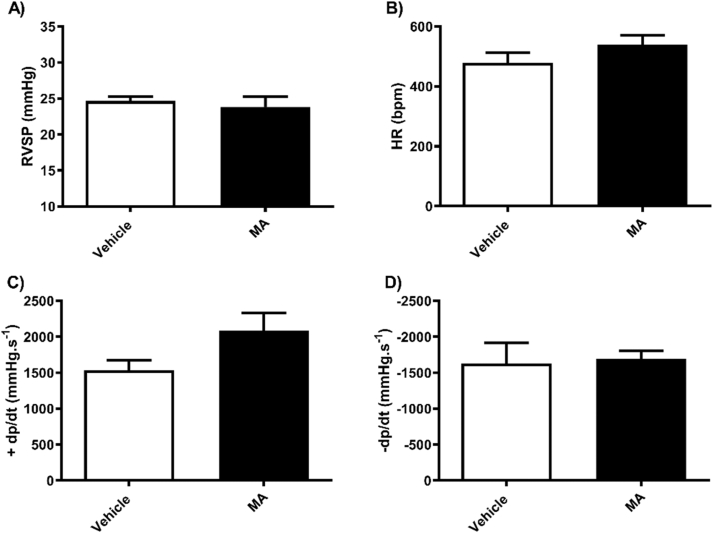
Methamphetamine (MA) or vehicle treatment in female mice had no effect on right ventricular systolic pressure (A) Heart rate (B) or right heart contractility (C and D). Results are expressed as mean ± standard error of the mean (S.E.M) (n = 5–6).

**Fig. 2:**
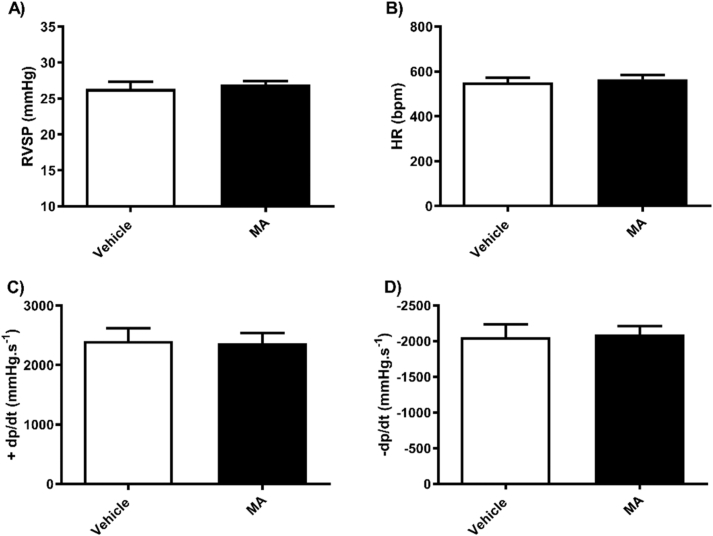
Methamphetamine or vehicle treatment in male mice had no effect on right ventricular systolic pressure (A) Heart rate (B) or right heart contractility (C and D). Results are expressed as mean ± standard error of the mean (S.E.M) (n = 8 each).

In female mice, no significant differences were observed in RVSP (Fig. 1A) and HR (Fig. 1B) between the vehicle and MA groups. In addition, MA treatment had no effect on right ventricular contractility; max dp/dt (Fig. 1C) and min dp/dt (Fig. 1D). Similarly, in the male mice, no significant differences were observed in RVSP (Fig. 2A), HR (Fig. 2B) and right ventricular contractility (Fig. 2C and D).

### 3.3 Effect of MA treatment on LVSP

**Fig. 3:**
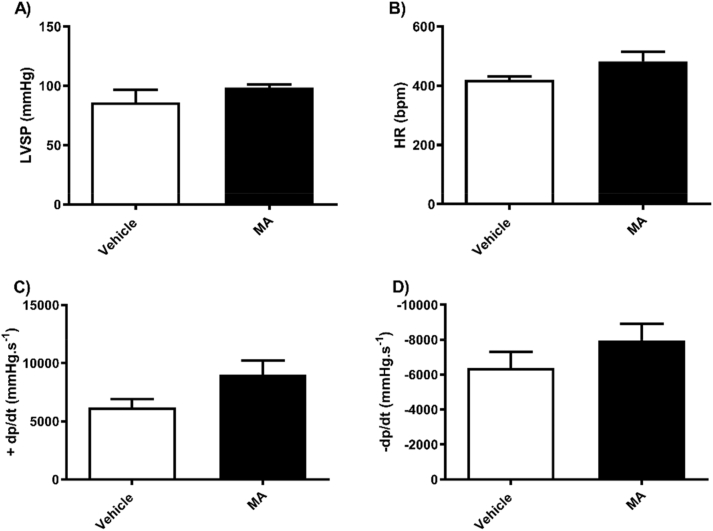
Methamphetamine or vehicle treatment in female mice did not affect left ventricular systolic pressure (A) Heart rate (B) or left heart contractility (C and D). Results are expressed as mean ± standard error of the mean (S.E.M) (n = 4–6).

**Fig. 4:**
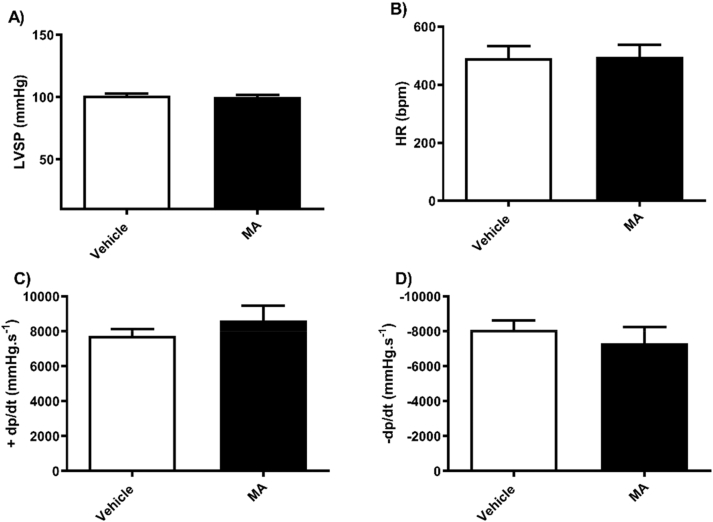
Methamphetamine or vehicle treatment in male mice did not affect left ventricular systolic pressure (A) Heart rate (B) or left heart contractility (C and D). Results are expressed as mean ± standard error of the mean (S.E.M) (n = 4–5).

In female mice, MA treatment did not have an effect on LVSP (Fig. 3A), HR (Fig. 3B), and left ventricular contractility; max dp/dt (Fig. 3C) and min dp/dt (Fig. 3D). In male mice, MA treatment did not affect LVSP (Fig. 4A), HR (Fig. 4B), nor left ventricular contractility (Fig. 4C and D).

### 3.4 Effect of MA treatment on gene expression in mouse lung

**Fig. 5:**
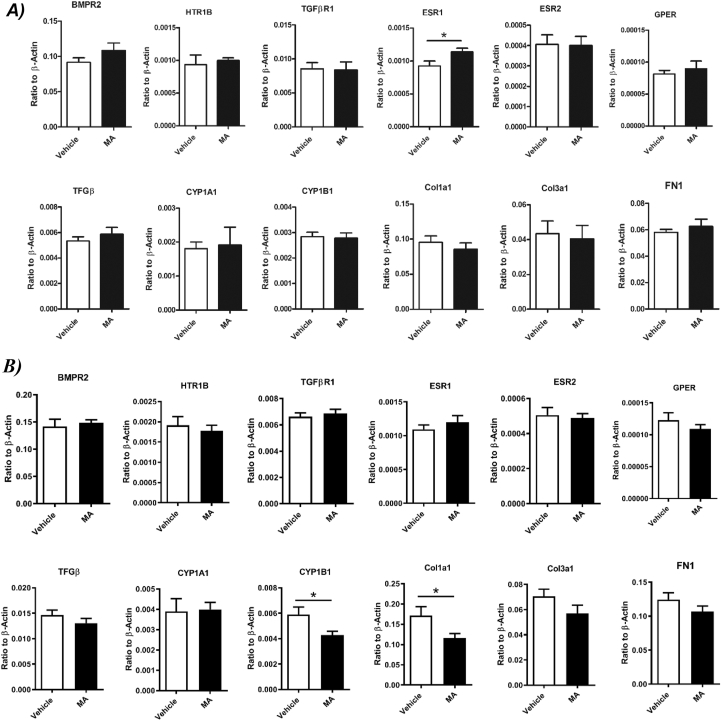
Expression of genes associated with PAH and fibrosis in lung tissue isolated from A) vehicle- (white) and methamphetamine- (black) treated female mice and B) vehicle- (white) and methamphetamine- (black) treated male mice. BMPR2 (Bone Morphogenetic Protein Receptor Type II), HTR1B (5-Hydroxytryptamine (Serotonin) Receptor 1B), TGFβR1 (Transforming Growth Factor Beta Receptor I), ESR 1 (Estrogen Receptor Alpha), ESR 2 (Estrogen Receptor Beta), GPER (G Protein-Coupled Estrogen Receptor 1), TGFβ1 (Transforming Growth Factor Beta 1), CYP1A1 (Cytochrome P450 Family 1 Subfamily A polypeptide 1),CYP1B1 (Cytochrome P450 Family 1 Subfamily B, Polypeptide 1), Col1a1 (collagen type I), Col3a1 (collagen type III), FN1 (fibronectin 1). Results are expressed as mean ± standard error of the mean (S.E.M) (n = 6). *P < 0.05 vs. vehicle group.

Gene expression of receptors and signaling pathway previously shown to contribute to PAH in female was assessed using qPCR. In female mouse lung tissue, except for an increase in estrogen receptor α gene expression (*ESR1)*, MA treatment did not affect gene expression of receptors shown to contribute to PAH such as *BMPR2* and *HTR1B*. No changes were observed in gene associated with estrogen metabolism (*CYP1A1* and *CYP1B1*), as well as genes associated with remodeling and fibrosis (*TGFβR1, cola1a1, col3a1* and *FN1*) (Fig. 5A). In the lung tissue isolated from male mice, no significant difference was observed in lung gene expression except for *CYP1B1* and Collagen I gene expression, which were significantly decreased in the male mice treated with MA (Fig. 5B).

### 3.5 Effect of MA treatment on gene expression in mouse RV

**Fig. 6:**
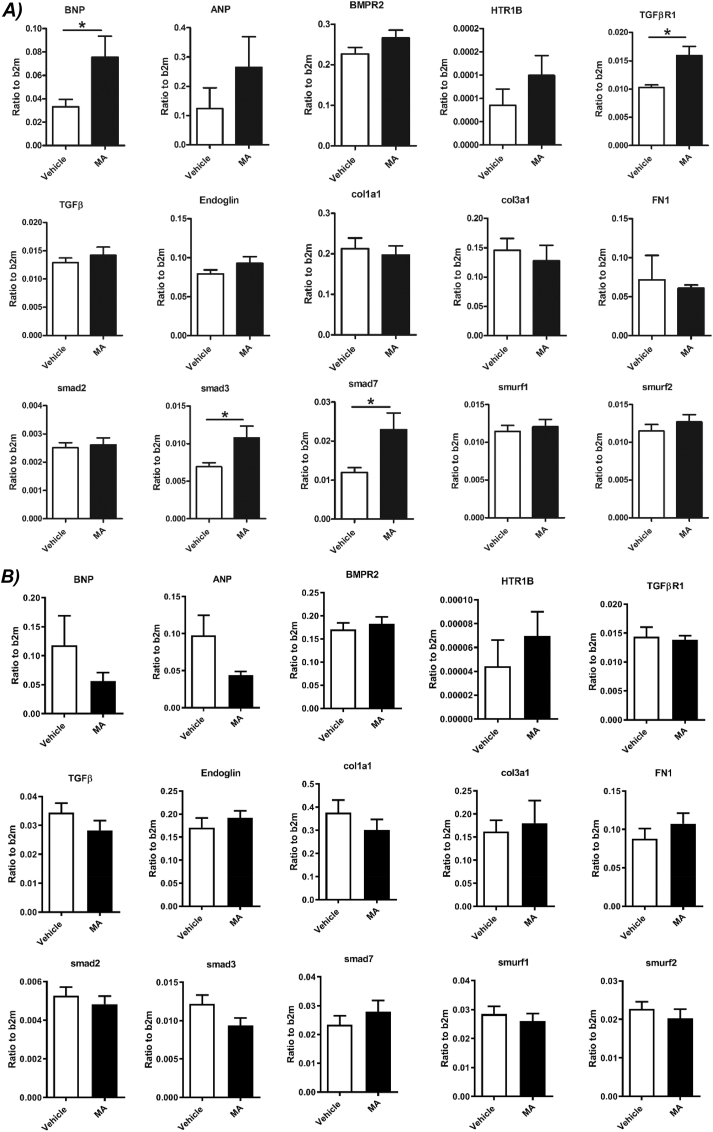
Expression of genes associated with PAH and fibrosis in the RV tissue isolated from A) vehicle- (white) and methamphetamine- (black) treated female mice and B) vehicle- (white) and methamphetamine- (black) treated male mice. ANP (natriuretic peptide type A), BNP (natriuretic peptide type B), BMPR2 (Bone Morphogenetic Protein Receptor Type II), HTR1B (5-Hydroxytryptamine (Serotonin) Receptor 1B), TGFβR1 (Transforming Growth Factor Beta Receptor I), TGFβ1 (Transforming Growth Factor Beta 1), Col1a1 (collagen type I), Col3a1 (collagen type III), FN1 (fibronectin 1), Smad 2 (SMAD family member 2), Smad 3 (SMAD family member 3), Smad 7 (SMAD family member 7), Smurf 1 (SMAD specific E3 ubiquitin protein ligase 1), Smurf 2 (SMAD specific E3 ubiquitin protein ligase 2). Results are expressed as mean ± standard error of the mean (S.E.M) (n = 6). *P < 0.05 vs. vehicle group.

In RV tissue isolated from female mice, MA treatment significantly increased the marker of right ventricular dysfunction, BNP. ANP gene expression tend to increase in RV from MA treated female mice, however, it was not significant. The expression of transforming growth factor beta receptor I (*TGFβR1*) and its downstream signaling genes (*Smad3* and *Smad7*) were significantly increased in RV isolated from MA-treated female mice. Gene expression of *BMPR2*, *HTR1B* and markers of fibrosis were not affected by MA (Fig. 6A). MA did not affect gene expression in RV isolated from male mice (Fig. 6B).

### 3.6 Effect of MA treatment on pulmonary vascular remodeling

**Fig. 7:**
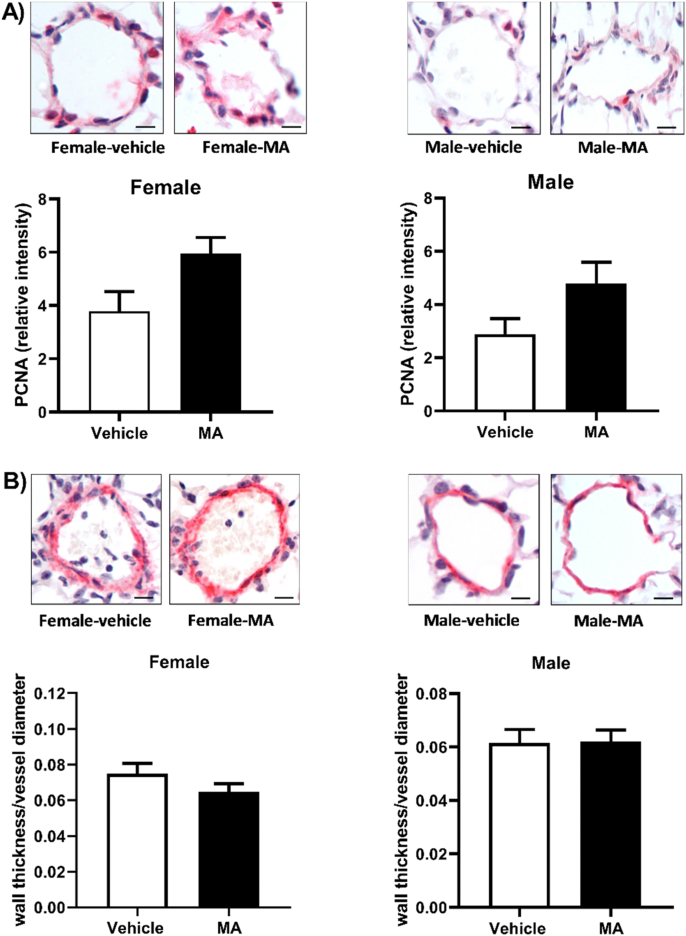
Pulmonary vascular immunostaining for A) the proliferation marker; proliferating cell nuclear antigen (PCNA) (n = 5–6), and for B) α-smooth muscle actin (α-SMA) (n = 6 each group). Results are expressed as mean ± standard error of the mean (S.E.M). Scale bars indicate 20 μm.

In both males and females, no significant pulmonary vascular remodeling was observed in lungs from MA-treated mice when compared to their corresponding vehicle-treated mice. In females, the percent of remodeled vessels was 3.68 ± 1.37% in vehicle vs. 4.24 ± 2.31% in MA group (P > 0.05), whilst in males it was 9.22 ± 4.07% in vehicle vs. 2.72 ± 0.73% in MA group (P > 0.05). Consistent with the absence of vascular remodeling, the expression of vascular PCNA was not affected by MA (Fig. 7A). In addition, MA had no effect on expression alpha-smooth muscle actin (Fig. 7B).

## 4 Discussion

The primary finding of this study is that, in female mice only, MA induces RVH associated with gene expression changes in the RV which may predispose development of experimental pulmonary hypertension in female mice. Those changes were not observed in male mice treated with MA. Clinically, females are more susceptible to PAH (Shapiro et al., 2012), and a recent clinical study demonstrated that the main factor associated with MA-induced PAH was female sex (Zhao et al., 2018). Furthermore, studies have reported that females have increased sensitivity to some behavioral effects of MA when compared to males. These include increased locomotor activity, highly likelihood of self-administer MA given free access to the drug, as well as higher vulnerability to relapse after period of forced abstinence (Ohia-Nwoko et al., 2017; Roth and Carroll, 2004; Ruda-Kucerova et al., 2015). Female rat hearts have increased sensitivity to ischemic injury after MA treatment compared to male rat hearts, suggesting a sex-dependent sensitivity to MA (Rorabaugh et al., 2016, 2017). Together, our results may suggest that the effects of MA on genes known to be associated with the development of PAH may play a role in MA-induced PAH in females (Zhao et al., 2018). In the present study, MA did not affect RVSP and LVSP in either male or female mice. In addition, the MA had no effect on pulmonary vascular remodeling or the expression of the markers of proliferation, PCNA and alpha smooth muscle actin. Our results agreed with previous studies showing that RV pressure is unaffected by MA in rats and mice (Chen et al., 2017a; Liu et al., 2013; Wang et al., 2013). While MA is among risk factors for pulmonary hypertension, it may act as a “second hit” to an underlying genetic (i.e. mutations) or non-genetic (i.e. Human Immunodeficiency Virus (HIV)) conditions (Ayala et al., 2012; Orcholski et al., 2017, 2018).

Our understanding of the mechanisms behind MA-associated hypertension is still lacking. In the vasculature, acute MA treatment has been shown to induce vasoconstriction in cerebral arterioles and cause blood brain barrier dysfunction (Kousik et al., 2011; Polesskaya et al., 2011; Seo et al., 2016). While the mechanisms of MA induced vasoconstriction are not well understood, studies have suggested the involvement of endothelin-1 or vascular trace amino acid receptor 1 (TAAR1) (Kevil et al., 2019). In a recent human study, chronic use of MA was associated with a decrease in endothelial-independent vasodilation in response to nitroglycerine, a nitric oxide donor (Nabaei et al., 2016). In the heart, MA use was shown to cause arrhythmia and cardiomyopathy. Most of studies focused on the effect of MA of the left ventricle, however, investigating the effect of MA on the cardiopulmonary system, especially the RV, becomes a necessity, since MA is now considered as definite risk factor for PAH.

Developing animal models of MA-induced PAH to mimic the human condition is difficult, due to the difference in rodent metabolism, as well as the frequency and the duration of MA use, as it takes years to develop PAH in MA abusers (Chen et al., 2017a). Despite these limitations however, even in the absence of increased RVSP, the female mice treated with MA exhibited an increase in RV hypertrophy (higher RV weight and Fulton index). This suggests that the RV changes are not secondary to increased pulmonary pressures but due to a direct effect of MA. Our results corroborate previous in vitro studies demonstrating increased cell size of rat cardiomyocytes in response to MA treatment (Maeno et al., 2000a, 2000b). This RV hypertrophy was associated with a significant increase in gene expression of BNP, which is regarded as a biomarker of right ventricular hypertrophy and dysfunction (Goncalves et al., 2017; Haworth, 2007). Moreover, studies have shown that plasma levels of BNP are proportional to the extent of RV dysfunction in pulmonary hypertension (Nagaya et al., 1998), and it was suggested that increased levels of BNP should be considered by cardiologists as an indication of a high risk of RV dysfunction (Mariano-Goulart et al., 2003). MA also has been shown to increase endothelin, angiotensin, serotonin and adrenergic signaling systems (Jiang et al., 2018; Liu et al., 2013; Seo et al., 2016; Sulzer et al., 2005; Wang et al., 2013), which are known vasoconstrictors and have been shown to contribute to the progression and pathogenesis of PAH (Iyinikkel and Murray, 2018). BNP is a vasodilator and is crucial in preventing myocardial hypertrophy and fibrosis (Haworth, 2007; Tamura et al., 2000), so the increased BNP may act as a compensatory and adaptive mechanism to counteract the effects of these vasoconstrictors and to reduce ventricular hypertrophy. Interestingly, while RV *TGF-β* transcript level was not affected by MA, gene expression of its receptor *TGF-βR1* and its downstream mediator smad3 were significantly increased in RV isolated from MA-treated female mice. TGF-β signaling has been shown to contribute to hypertrophy, apoptosis and fibrosis in cardiomyocytes, which can lead to myocardial infarction (Dobaczewski et al., 2011; Euler, 2015). The Smad ubiquitin regulatory factors 1 and 2 (Smurf1 and smurf2), which negatively regulate TGF-β signaling by inducing TGF-β receptor degradation (Huang and Chen, 2012; Koganti et al., 2018), were not affected by MA. RV Smad7 gene expression was also increased by MA in the female mice. Smad7 is a negative regulator of TGF-β signaling and so this may be a compensatory mechanism. Indeed, TGF-β has been shown to induce Smad7 gene expression (Afrakhte et al., 1998; Quezada et al., 2012; Zhao et al., 2000). Together, our data suggest that TGF-β may have induced increase in Smad7 expression, which could act as a negative feedback mechanism to counteract the increased TGF-βR1 signaling in the hypertrophic RV. The expression of receptors associated with pulmonary hypertension and RV remodeling such as receptor (*HTR1B*), BMPR2 and endoglin (Gore et al., 2014; Hautefort et al., 2019; Hood et al., 2017; Keegan et al., 2001; Wallace et al., 2015) were not affected by MA. Additionally, we did not see an effect of MA on expression of fibrosis genes such as *col1a1*, *col3a1* and *FN1*. In male mice, there were no RV changes in RV gene expression after MA treatment consistent with the absence of MA-induced RV structural changes in male mice. Our data suggest that, in females, MA induces early changes in gene expression in the RV that precedes any cardiac dysfunction.

MA did not affect the expression of fibrotic genes in the female mouse lung. Our laboratory and others have extensively investigated the role and the contribution of estrogen, estrogen receptors and estrogen-metabolizing enzymes to the pathogenesis and progression of PAH (Chen et al., 2017b; Dean et al., 2018; Hood et al., 2017; Johansen et al., 2016; Mair et al., 2014; Wallace et al., 2015; White et al., 2012; Wright et al., 2015). MA did not affect the estrogen-metabolizing enzymes CYP1A1 and CYP1B1 in lungs from female mice. However, MA induced an increase in estrogen receptor α (*ESR1*) gene expression, but not gene expression of estrogen receptor β (*ESR2*) or the G-protein coupled estrogen receptor (*GPER*). Previous studies have shown that ERα gene expression is increased in the lung and pulmonary artery smooth muscle cells isolated from PAH patients (Rajkumar et al., 2010; Wright et al., 2015), suggesting that MA may contribute to PAH at an early stage by increasing estrogen signaling through ERα upregulation. MA did not affect gene expression in male lungs except for a decrease in both *CYP1B1* and *Col1a1* genes. We can only speculate that these changes in *CYP1B1* and *Col1a1* genes may act as mechanisms to offset the negative effect of MA on the lung. In fact, we have previously shown that genetic deletion of CYP1B1 in mice attenuates hypoxia-mediated increased RVSP in male but not in female mice, suggesting that PAH-associated mechanisms differ between males and females (White et al., 2012).

Recent studies have suggested that MA can induce pulmonary vascular remodeling (Chen et al., 2017a; Liu et al., 2013; Wang et al., 2013). However, in the present study, we did not see a significant remodeling of pulmonary vessels in both male and female mice. This may be a result of different experimental protocols, for example using a lower MA dose (0.5 mg/kg vs. 10 mg/kg), the length of the study (3 weeks vs. 5 weeks) or the experimental design (use of MA injection alone vs. MA injection in combination with hypoxia).

## 5 Conclusion

To our knowledge, this study is the first to investigate the effect of MA in female mice. We demonstrate effects of MA on the lung and the RV in female mice, which we did not observe when investigating the effect of MA in male mice. The increased hypertrophy and TGF-β receptor signaling in the RV and increased ERα signaling in the lung may increase the susceptibility of the female mice to development of PAH. MA-associated PAH was shown to be a severe and progressive form of PAH with a poor outcome (Zamanian et al., 2018). Our study is consistent with others showing that MA induces gene expression changes and structural changes (RV hypertrophy and vascular remodeling) in the heart and the lung. This may explain the increased susceptibility of female MA abuser to develop PAH as well as the poor outcome of MA-associated PAH compared to idiopathic PAH.

Although our study suggests potential mechanisms that may lead to more progressive form of PAH and worst clinical outcome in MA-associated PAH, there are limitations to the use of mouse as the mouse experimental models of pulmonary hypertension demonstrate only moderate pulmonary hypertension.

## Authors contributions

HL and MRM conceived and designed research; HL and MN performed experiments and analyzed data; HL drafted the manuscript; HL and MRM revised and edited the manuscript.

## Funding

This study was supported by a programme grant from 10.13039/501100000274British Heart Foundation (RG/16/2/32153).

## CRediT authorship contribution statement

**Hicham Labazi:** Conceptualization, Methodology, Formal analysis, Writing – original draft, Writing – review & editing. **Margaret Nilsen:** Methodology, Analysis. **Margaret R. MacLean:** Conceptualization, Funding acquisition, Writing – review & editing.

## Declaration of competing interest

The authors declare no Conflict of Interest.
